# Supplementary material for: Inflammation Regulation via an Agonist and Antagonists of α7 Nicotinic Acetylcholine Receptors in RAW264.7 Macrophages
Source: Mar Drugs. 2022 Mar 9;20(3):200. doi: 10.3390/md20030200 (PMC8955479; doi:10.3390/md20030200)
Supplement: Supplementary file 1 [file marinedrugs-20-00200-s001.zip › marinedrugs-1605482-supplementary.pdf]

Supplementary Material

Inflammation Regulation via an Agonist and Antagonists of α7 Nicotinic Acetylcholine Receptors in RAW264.7 Macrophages

Yao Tan[1], Zhaoli Chu[2], Hongyu Shan[1], Dongting ZhangSun[2], Xiaopeng Zhu[1,*], Sulan Luo[1,2*]

1 Medical School, Guangxi University, Nanning, 530004, China; tanyao971006@163.com (Y.T.); dlhy34@163.com (H.S.)

2 Key Laboratory of Tropical Biological Resources of Ministry of Education, School of Pharmaceutical Sciences, Hainan University, Haikou, 570228, China; 220100700210003@hainanu.edu.cn (Z.C.); zhangsundt@163.com (D.ZS.)

* Correspondence: biozxp@163.com (X.Z.); Sulan2021@gxu.edu.cn (S.L.)

Table S1. Primers Table.

nAChRs	NCBI number	5′→3′	Tm(°C)	Length (bp)
Chrna-3	NM_110834	F: CTACGACAAGGCAAAGATCG	53	134
		R: CCTCACAGCAGTTGTACTTG		
Chrna-4	NM_11438	F: CATCTCGGTGCTGCTTTCTC	55	164
		R: GGTGGTGTACATTGAGCACG		
Chrna-5	NM_110835	F: CACTCAGAGAGAAGAAGCCG	55	166
		R: CATCCGATCGAGAACTTGGG		
Chrna-6	NM_11440	F: GACTTCCAGGTCGAAGGCAA	55	179
		R: AGGTCGATTTCTGCCTTGTC		
Chrna-7	NM_11441	F: TGCAGTGGAACATGTCTGAG	54	153
		R: AATGCCCAGATGCATTCACC		
Chrna-9	NM_231252	F: GCCGTGAAGAACGTCATCTC	55	142
		R: GCGGAGCGAGGAACGATATG		
Chrna-10	NM_504186	F: CAAGTGCCTTGAGACCAGTG	55	213
		R: GCCACACTAGACTGCTGGGA		
Chrnb-2	NM_11444	F: GGTGACTGTACAGCTCATGG	55	167
		R: GCTTAGAAGGGAGTCGGACT		
Chrnb-3	NM_108043	F: CAATGAAGTTCGGATCCTGG	53	148
		R: AAGCCTTCTCTTCTGTTGCC		
Chrnb-4	NM_108015	F: GGACCTATGACCACACAGAG	56	157
		R: AAGTCATAGGTCACGTCCAC		
Tnf-α	Y00467	F: GAACTGGCAGAAGAGGCAC	55	137
		R: GTAGACAGAAGAGCGTGGTG		
Il-1β	NM_008361	F: ACTTCGCAGCAGCACATC	55	120
		R: GCTCATGTCCTCATCCTGGA		
Il-6	M24221	F:TGTATGAACAACGATGATGCACTTG	55	147
		R:AGGTAGCTATGGTACTCCAGAAGAC		
β-actin	NM_007393	F: CTGTATTCCCCTCCATCGTGG	55	279
		R:TCTGGGTCATCTTTTCACGGT		
Gapdh	DQ403054	F: CATCACTGCCACCCAGAAG	55	137
		R:CGTTCAGCTCTGGGATGAC		

After finding the corresponding gene sequence on NCBI, the ORF region was found through EditSeq software, primers were designed in Snap Gene software, and blast was performed in NCBI to confirm the specificity of the primers.

Table S2. Statistical table of expression of nAChR subunits in RAW264.7

Number	α3	α4	α5	α6	α7	α9	α10	β2	β3	β4
1	+	+	+	+	+	+	+	+	+	+
2	+	+	+	+	+	+	+	+	+	+
3	+	+	+	+	+	+	+	+	+	+
4	+	+	+	+	+	+	+	+	+	+
Total	4	4	4	4	4	4	4	4	4	4

Cultured four different batches of mouse monocyte macrophages RAW264.7 to determine the expression of nAChR subunits respectively.

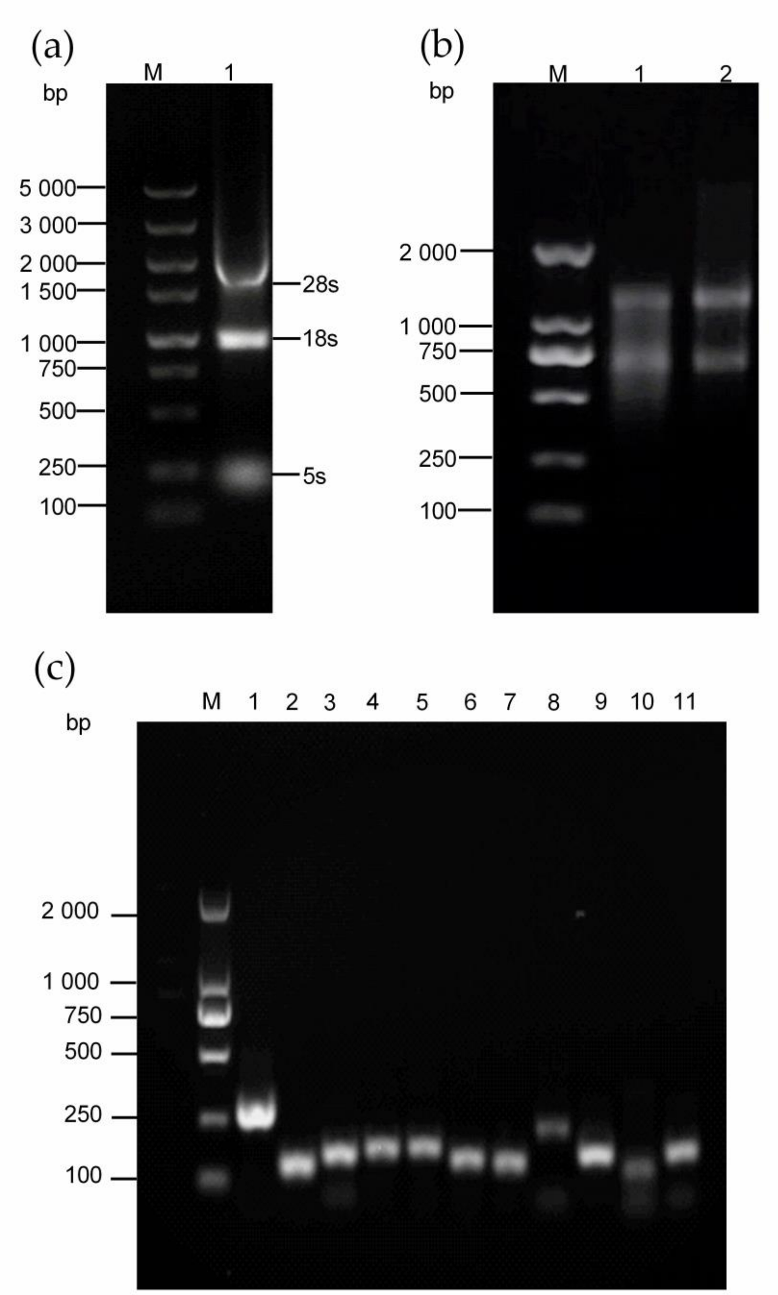

Figure S1. Agarose gel electrophoresis for identification of nAChR subunits. After the cells were cultured for 24 hours, the total RNA was extracted and reversed transcription into cDNA, and PCR amplification was performed using cDNA as a template to determine the expression of nAChR subunits in RAW264.7. (**a**): 0.8% Agarose gel electrophoresis analysis of RAW 264.7 total RNA, M: DL5 000 RNA Marker; (**b**): 1% Agarose gel electrophoresis analysis of RAW 264.7 cDNA, M: DL2 000 DNA Marker, 1: control 2: cDNA; (**c**): 1% garose gel electrophoresis analysis of RAW 264.7 PCR product M: DL2 000 DNA Marker , 1-11: *Gapdh*, α3, α4, α5, α6, α7, α9, α10, β2, β3, β4.

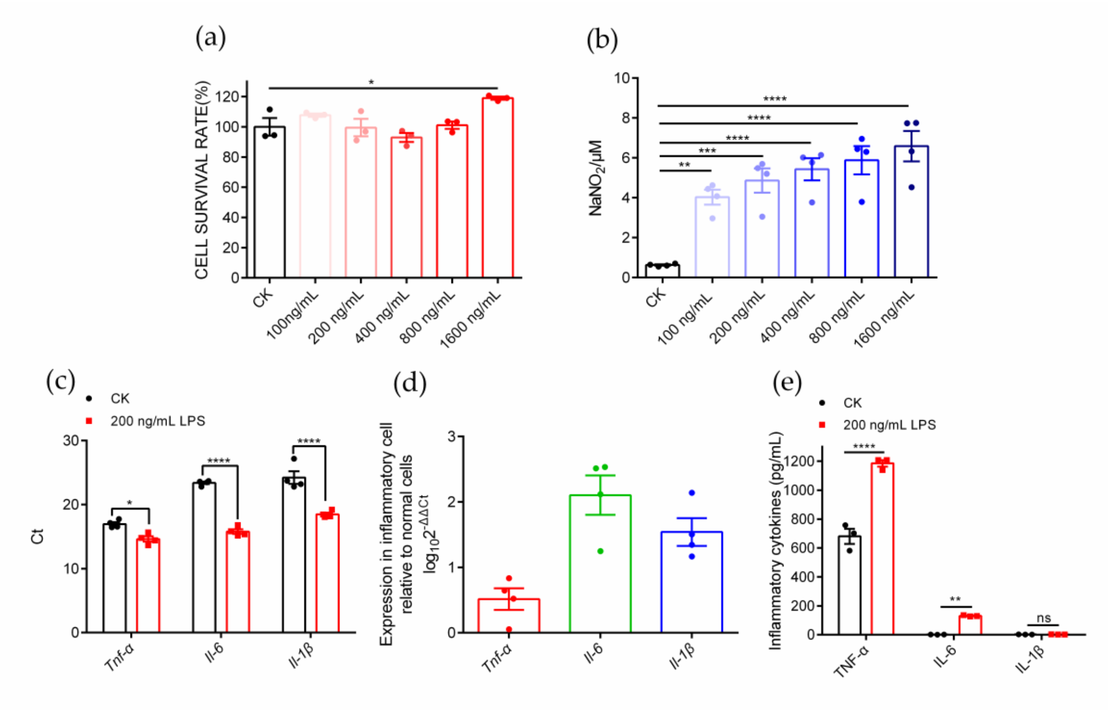

Figure S2. Construction of RAW264.7 inflammation model. After normal cells were cultured for 24 h, LPS (200 ng/mL) was administered to induce 12-16 h, and the inflammatory response was determined from the upregulation of NO level, proinflammatory cytokine(*Tnf-α*, *Il-6*, *Il-1β*)gene and protein levels. (**a**): CCK8 experiment is used to determine the appropriate induction concentration for a series of concentration gradients of LPS; (**b**): NO detection for a series of concentration gradients of LPS is used to determine the appropriate induction concentration; (**c**): Comparison of Ct values of inflammatory cytokines in normal cells and inflammatory cells; (**d**): Upregulation of the expression of proinflammatory cytokines mRNA in inflammatory cells compared with normal cells; (**e**): Upregulation of proinflammatory cytokines protein expression in inflammatory cells compared with normal cells. mRNA expression of proinflammatory cytokines were determined by using qPCR, Protein expression of proinflammatory cytokines were determined by ELISA kit. ns, not significant or $p > 0.05$; *, $p \leq 0.05$; **, $p \leq 0.01$; *** , $p \leq 0.001$; ****, $p \leq 0.0001$ compared to the control groups , by one-way ANOVA followed by the Dunnett's multiple comparisons test, the error bars in all figures indicate SEM(n=3-5).
